# Diffuse Glomerular Nodular Lesions in Diabetic Pigs Carrying a Dominant-Negative Mutant Hepatocyte Nuclear Factor 1-Alpha, an Inheritant Diabetic Gene in Humans

**DOI:** 10.1371/journal.pone.0092219

**Published:** 2014-03-19

**Authors:** Satoshi Hara, Kazuhiro Umeyama, Takashi Yokoo, Hiroshi Nagashima, Michio Nagata

**Affiliations:** 1 Department of Kidney and Vascular Pathology, University of Tsukuba, Tsukuba, Japan; 2 Meiji University International Institute for Bio-Resource Research, Kawasaki, Japan; 3 Division of Nephrology and Hypertension, Department of Internal Medicine, The Jikei University School of Medicine, Tokyo, Japan; 4 Division of Rheumatology, Department of Internal Medicine, Kanazawa University of Graduate School of Medicine, Kanazawa, Japan; University of Florida, United States of America

## Abstract

Glomerular nodular lesions, known as Kimmelstiel-Wilson nodules, are a pathological hallmark of progressive human diabetic nephropathy. We have induced severe diabetes in pigs carrying a dominant-negative mutant hepatocyte nuclear factor 1-alpha (HNF1α) P291fsinsC, a maturity-onset diabetes of the young type-3 (MODY3) gene in humans. In this model, glomerular pathology revealed that formation of diffuse glomerular nodules commenced as young as 1 month of age and increased in size and incidence until the age of 10 months, the end of the study period. Immunohistochemistry showed that the nodules consisted of various collagen types (I, III, IV, V and VI) with advanced glycation end-product (AGE) and *N* ^ε^-carboxymethyl-lysine (CML) deposition, similar to those in human diabetic nodules, except for collagen type I. Transforming growth factor-beta (TGF-β) was also expressed exclusively in the nodules. The ultrastructure of the nodules comprised predominant interstitial-type collagen deposition arising from the mesangial matrices. Curiously, these nodules were found predominantly in the deep cortex. However, diabetic pigs failed to show any of the features characteristic of human diabetic nephropathy; e.g., proteinuria, glomerular basement membrane thickening, exudative lesions, mesangiolysis, tubular atrophy, interstitial fibrosis, and vascular hyalinosis. The pigs showed only Armanni-Ebstein lesions, a characteristic tubular manifestation in human diabetes. RT-PCR analysis showed that glomeruli in wild-type pigs did not express endogenous HNF1α and HNF1β, indicating that mutant HNF1α did not directly contribute to glomerular nodular formation in diabetic pigs. In conclusion, pigs harboring the dominant-negative mutant human MODY3 gene showed reproducible and distinct glomerular nodules, possibly due to AGE- and CML-based collagen accumulation. Although the pathology differed in several respects from that of human glomerular nodular lesions, the somewhat acute and constitutive formation of nodules in this mammalian model might provide information facilitating identification of the principal mechanism underlying diabetic nodular sclerosis.

## Introduction

Diabetic nephropathy is the leading cause of end-stage renal disease [1], [2]. Glomerular nodular lesions, known as Kimmelstiel-Wilson nodules, are a pathological hallmark of human diabetic nephropathy. In 1936 Kimmelstiel and Wilson first described intercapillary glomerulosclerosis as a sign of advanced diabetic glomerular changes [3]. The presence of glomerular nodular lesions is known to be associated with poor renal outcome [4], [5].

Although investigated extensively, the morphogenesis of diabetic glomerular nodules remains to be determined. One major reason for this is a lack of animal models that accurately represent the nodules typically present in humans. Some rodent models of diabetes show segmental mesangial expansion and glomerular basement membrane (GBM) thickening, but few exhibit distinct glomerular nodular lesions [6]. To date, the four representative diabetic rodent models with glomerular nodules are: endothelial nitric oxide synthase (eNOS) knockout *db/db* mice [7], receptor for advanced glycation end products (RAGE)/megsin/inducible nitric oxide synthase (iNOS) overexpressing transgenic mice [8], monocrotaline-treated Otsuka Long-Evans Tokushima Fatty (OLETF) rats [9] and BTBR *ob/ob* mice [10]. eNOS knockout *db/db* mice developed focal nodular glomerulosclerosis at 26 weeks of age [7]. RAGE/megsin/iNOS overexpressing transgenic mice also showed nodular-like lesions in 30–40% of glomeruli at 16 weeks of age [8]. Monocrotaline-treated OLETF rats showed a few nodular-like lesions at 50 weeks of age [9]. BTBR *ob/ob* mice showed diffuse but rare nodular mesangial sclerosis at 20 weeks of age [10]. These rodent models suggest that diabetic conditions in rodents do not lead to reproducible formation of diffuse glomerular nodular lesions. In addition, although two diabetic pig models—streptozotocin-induced diabetic pigs and *INS* ^C94Y^ transgenic pigs—were created, both failed to reproduce diabetic kidney manifestations [11], [12], [13]. Thus, it may be more appropriate to create a diabetic mammalian model with the same genetic mutations present in human diabetes that exhibits diabetic renal complications similar to those in humans.

In humans, several forms of diabetes are associated with genetic mutations. Maturity-onset diabetes of the young type-3 (MODY3) is an early onset, non-insulin-dependent form of diabetes characterized by autosomal-dominant inheritance [14]. Those suffering from MODY3 have insufficient insulin secretion, resulting in a similar pathophysiology to that seen in human type-2 diabetes [14], [15]. Hepatocyte nuclear factor 1-alpha (HNF1α) is the transcription factor believed to be responsible for MODY3 [14], [15]. It is expressed in the liver, pancreas, proximal tubules, stomach, and small intestine [15], [16], [17]. The most common mutation in the HNF1α gene is the result of a cytosine (C) nucleotide insertion into a poly-C tract around codon 291 (designated as P291fsinsC), which causes frameshift-mutation-mediated deletion of the transactivation domain [14], [15].

We have successfully created diabetic pigs carrying the dominant-negative mutant HNF1αP291fsinsC gene that is responsible for severe hyperglycemia with decreasing numbers of pancreatic beta cells [18]. Using these transgenic animals, in the present study we investigated the sequence of morphological events that leads to glomerular nodular lesions in diabetic nephropathy based on the human MODY3 gene. We expected the components and processes of glomerular nodular lesions in diabetic pigs to resemble those in human diabetic nephropathy.

## Materials and Methods

### Animals

All animal experiments were approved by the Institutional Animal Care and Use Committee of Meiji University (IACUC-09-006). As described previously, focus was on the use of transgenic pigs carrying a dominant-negative mutant HNF1α gene [18]. In short, a transgenic pig carrying an expression vector for the mutant human HNF1α cDNA (HNF1αP291fsinsC) was used. The transgene construct consisted of the enhancer for an immediate-early gene of human cytomegalovirus, followed by a porcine insulin promoter, the human HNF1αP291fsinsC cDNA, a SV40 poly-adenylation signal and a chicken β-globin insulator. Transgenic pigs carrying this cDNA were produced as reported elsewhere [19].

### Study protocol

One transgenic and three wild-type pigs were used for biochemical and histological analyses through kidney biopsy. Tests were conducted at monthly intervals until the animals were 10 months of age. For histological analyses, autopsy of additional three transgenic and three wild-type pigs was conducted at 19 weeks of age.

### Biochemical analysis

Serum and urine were collected each month after birth until completion of the study. The following biochemical parameters were measured: blood urea nitrogen, creatinine, plasma glucose, total protein, total cholesterol, triglycerides, aspartate aminotransferase, alanine aminotransferase and 1,5-anhydroglucitol. Urine was also analyzed in terms of total protein/creatinine and albumin/creatinine.

### Histochemistry of renal sections

For kidney biopsy, the animals were anesthetized by an intramuscular injection of ketamine (11 mg/kg, Fujita Pharmaceutical Co., Ltd., Tokyo, Japan), with isoflurane (DS Pharma Animal Health Co., Ltd., Osaka, Japan) inhalation for maintenance. After the kidney location was confirmed using an ultrasonic pulse-echo technique, specimens were obtained using a Bard Monopty disposable biopsy needle (18 G×20 cm, Bard Biopsy Systems, Tempe, AZ, USA). Kidney specimens were fixed with 4% paraformaldehyde for paraffin sections or 2% glutaraldehyde for electron microscopy. For kidney autopsy, the animals were anesthetized by an intramuscular injection of 1% mafoprazine (0.5 mg/kg, DS Pharma animal Health Co., Ltd.) and intravenous injection of pentobarbital (Kyoritsu Seiyaku Corporation, Tokyo, Japan). After the animals were sacrificed by exsanguination through cutting cervical artery under anesthesia, kidney tissues were dissected and fixed with 4% paraformaldehyde for paraffin sections.

Paraffin sections were processed for periodic acid–Schiff (PAS) staining, periodic acid–methenamine-silver (PAM) staining, Masson's trichrome (MT) staining and immunostaining. Specific primary antibodies were as follows: mouse anti-collagen I antibody (1∶50; Abcam, Cambridge, UK), rabbit anti-collagen III antibody (1∶400; Abcam), rabbit anti-collagen IV antibody (1∶50; Abcam), mouse anti-collagen V antibody (1∶50; Abcam), rabbit anti-collagen VI antibody (1∶50; Abcam), rabbit anti-advanced glycation end products (AGE) antibody (1∶250; Abcam), mouse anti-*N* ^ε^-carboxymethyl-lysine (CML) antibody (1∶500; TransGenic Inc., Ltd., Kumamoto, Japan) and rabbit anti-transforming growth factor beta-1 (TGF-β1) (V) antibody (1∶100; Santa Cruz Biotechnology Inc., Santa Cruz, CA, USA). For immunostaining, antigen retrieval was performed using a microwave (10 mM citrate buffer; pH 6.0) (collagen I) or 100 μg/mL proteinase K (collagen III, IV, V, and VI). Thereafter, primary antibodies were incubated in an EnVision labeled polymer-HRP (Dako, Glostrup, Denmark) or Histofine kit (Nichirei Bioscience Inc., Tokyo, Japan) followed by reaction with peroxidase-conjugated streptavidin (Nichirei). Peroxidase activity was visualized using a liquid diaminobenzidine substrate (Dako). Hematoxylin was used to stain nuclei.

### Distribution of glomeruli with nodular lesions

To estimate the prevalence of glomeruli with nodular lesions between the superficial and deep cortexes, sections representing the entire depth of the cortex were subdivided into three zones of equal width: the superficial, middle and deep cortex. The proportion of glomeruli with nodules in each sample was calculated and compared between the superficial and deep cortexes in autopsy specimens of transgenic and wild-type pigs at 19 weeks of age (60–240 glomeruli per kidney per animal).

### Measurement of glomerular tuft area

To estimate glomerular tuft area between the superficial and deep cortexes, sections representing the entire depth of the cortex were subdivided into three zones of equal width: the superficial, middle and deep cortex. The glomerular tuft area in each sample was calculated using NanoZoomer 2.0-RS (Hamamatsu Photonics K.K., Hamamatsu, Japan) and compared between the superficial and deep cortexes in autopsy specimens of transgenic and wild-type pigs at 19 weeks of age (69–393 glomeruli per kidney per animal).

### Thickness of the glomerular basement membrane

In biopsy specimens of pigs at 4 weeks and 5 months of age, 2% glutaraldehyde-fixed kidney cortex tissue was visualized by transmission electron microscopy. GBM thickness was estimated by measurements at five random capillaries in one glomerulus per animal. In each capillary, a series of five photographs were taken at 12,000× magnification, a grid was overlaid on the photograph, and GBM thickness was measured at the points intersecting the grid, with the exception of paramesangial areas. This method is a modified version of that of Hudkins, *et al.* [10].

### Glomerular isolation, RNA isolation and reverse transcription PCR (RT-PCR)

To evaluate HNF1α or HNF1β expression, RT-PCR was performed using isolated glomeruli from one wild-type pig at 4 weeks of age. The animal was anesthetized using isoflurane (DS Pharma Animal Health Co., Ltd.) and perfused with phosphate-buffered saline (PBS). The kidneys, liver and heart were then removed. Using the renal artery, the kidneys were perfused with a 1 mg/mL iron powder in PBS. They were then minced into 1-mm^3^ pieces and passed through a 100-μm cell strainer. Finally, glomeruli containing the iron powder were isolated using a magnetic particle concentrator. Total RNA was extracted from the isolated glomeruli, liver, and heart using the RNeasy Mini Kit (Qiagen, Hilden, Germany). RNA was quantified using a Nanodrop 1000 spectrophotometer (Thermo Fisher Scientific K.K., Rockford, IL). Total RNA (1 μg) was reverse-transcribed using the Thermoscript RT-PCR System (Life Technologies Corporation, Carlsbad, CA, USA) into first-strand cDNA. Then, 10 ng of cDNA template and 0.25 mmol/l of sequence-specific primers were used to perform RT-PCR. Primer sequences (5′ to 3′) were as follows: HNF1α forward: CACAGTCTGCTGAGCACAGA

HNF1α reverse: TTGGTGGTGTCGGTGATGAG

HNF1β forward: AGAGGGAGGCCTTAGTGGAG

HNF1β reverse: GAGAGGGGCGTCATGATGAG

The liver and heart were used as positive and negative controls, respectively.

### Statistical analysis

Mann-Whitney *U* tests using StatView-J 5.0 (Adept Scientific, Acton, MA, USA) were performed for comparison of the glomerular nodular distribution and glomerular tuft area. Data are shown as means ± standard errors (SE). *P*-values were calculated from the data. Statistical significance was considered at *p*-values < 0.05.

## Results

### Transgenic pigs carrying a dominant-negative mutant HNF1α gene showed severe diabetic mellitus

The biochemical parameters of a single transgenic pig were compared with those of three wild-type pigs over a 10-month period (Table 1). Body weight was lower in transgenic pigs than in wild-type pigs (Figure S1A). In transgenic pigs, the plasma glucose levels were elevated to 22.2–33.3 mmol/L as early as 11 days after birth. This hyperglycemia persisted until 10 months of age (Figure S1B). 1,5-Anhydroglucitol, which reflects the increase in plasma glucose levels during the past several days, was at low levels, indicating severe diabetes mellitus (Figure S1C). In 1-month-old pigs, total cholesterol was high, but decreased after 2 months of age. In contrast, triglycerides were elevated throughout the lifespan of the pigs, which is a symptom also observed in humans with diabetic mellitus. However, serum creatinine levels were within the normal range and no proteinuria was detected in transgenic pigs until 10 months of age.

**Table 1:** Analysis of biochemical parameters in transgenic (Tg) and wild-type (WT) pigs at age 1, 5 and 10 months.

	1 month old		5 months old		10 months old	
	Tg (n = 1)	WT (n = 3)	Tg (n = 1)	WT (n = 3)	Tg (n = 1)	WT (n = 1)
Blood urea nitrogen (mmol/l)	13.6	2.71±0.43	10.8	5.07±0.43	9.35	4.53
Plasma glucose (mmol/l)	33.3	6.11±0.03	>33.3	5.87±0.40	26.0	5.51
Creatinine (μmol/l)	53.0	61.9±0.0	35.4	88.4±8.8	26.5	115
Total protein (g/l)	54.0	48.0±1.0	63.0	62.0±0.0	72.0	68.0
Total cholesterol (mmol/l)	11.6	2.95±0.18	1.86	2.00±0.05	1.09	1.66
Triglycerides (mmol/l)	1.69	0.26±0.03	>5.60	0.50±0.10	4.35	0.17
Asperate aminotransferase (IU/l)	23.0	43.0±6.4	128	21.7±1.0	51.0	21.0
Alanine aminotransferase (IU/l)	38.0	31.0±2.3	68.0	33.3±0.7	62.0	32.0
1,5-anhydroglucitol (μg/ml)	2.8	8.8±0.3	1.1	9.6±0.7	2.5	6.7
Urinary protein/creatinine (g/gCr)	<0.20	0.24*	0.72	0.47±0.31	0.45	<0.20
Urinary albumin/creatinine (g/gCr)	<0.10	0.16*	1.41	0.77±0.59	0.23	<0.1

Aberrations: Tg  =  transgenic pigs; WT  =  wild-type pigs; *: n = 1.

### Transgenic pigs exhibited characteristic diffuse glomerular nodular lesions

**Figure 1:**
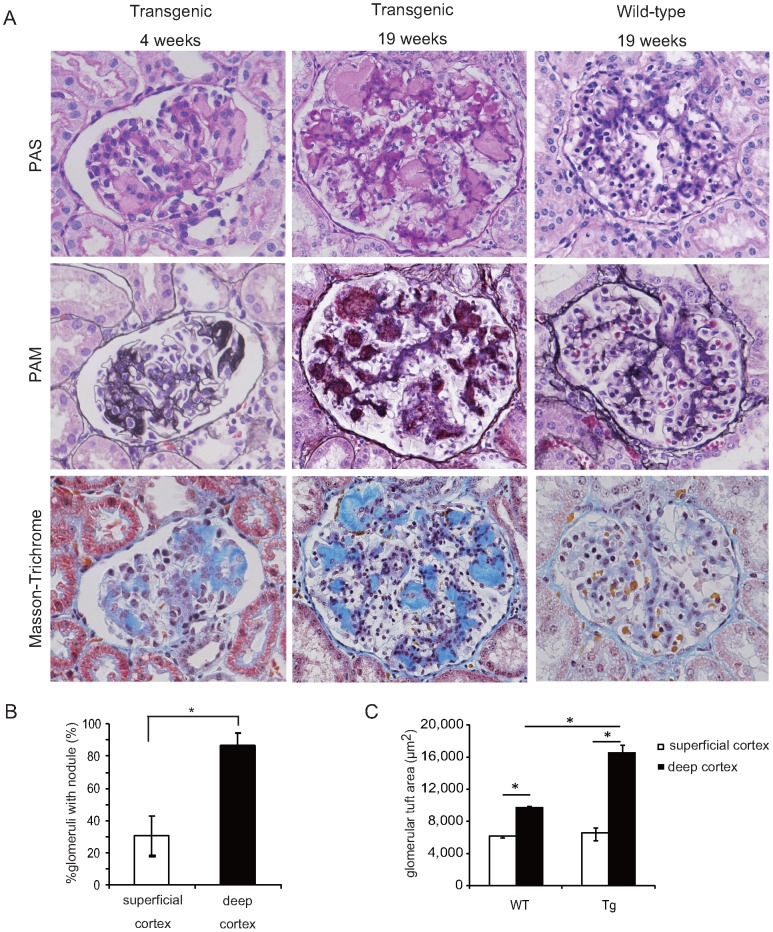
Renal pathological findings at age 4 and 19 weeks in transgenic pigs. **A**) In transgenic pigs, mesangial expansion commenced as early as 4 weeks. At 19 weeks, distinct glomerular nodules had formed. Magnification: 400×. **B**) The number of glomeruli with nodules as a fraction of the total number was compared between the superficial cortex and deep cortex. **C**) Glomerular tuft area in superficial and deep cortexes was compared between wild-type pigs and transgenic pigs. Transgenic pigs; n = 3, wild-type pigs; n = 3. *P<0.05. WT  =  wild-type pigs; Tg  =  transgenic pigs.

Kidney autopsy revealed distinct glomerular nodular lesions at age 19 weeks in all three transgenic pigs (Figure 1A). These nodules were diffuse and acellular, consisting of abnormal matrices. Matrices were slightly evident by PAS staining, strongly by PAM staining, and appeared as a distinct blue color by MT staining. This staining pattern points to the abundance of collagen fibers in the nodules. Numerous nodules formed within an individual glomerulus and were distributed throughout with no discernible pattern. However, more were present in the deep cortex than in the superficial cortex (86.6±7.73 *vs*. 30.6±12.2%) (*p* = 0.0495; Figure 1B). Additionally, the glomerular tuft area in the deep cortex was significantly larger in transgenic pigs than in wild-type pigs (16,566±983 *vs*. 9,694±224 μm^2^; *p* = 0.0495), but was not significantly different in the superficial cortex (6,616±588 *vs*. 6,166±80 μm^2^; *p* = 0.8273; Figure 1C). This unique distribution of nodules and glomerular tuft size suggested that glomerular hyperfiltration might contribute to formation of nodules in transgenic pigs.

**Figure 2:**
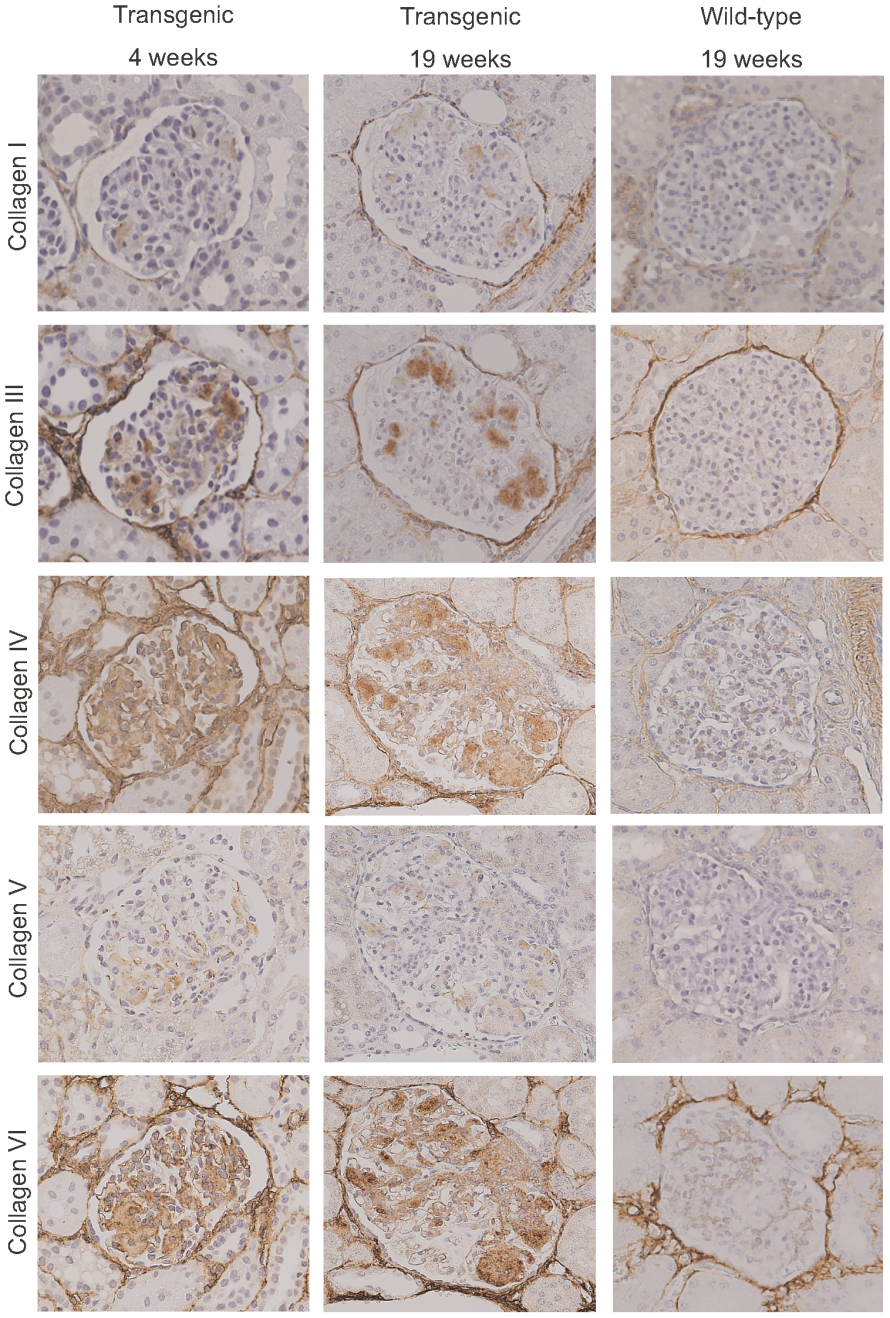
Immunostaining for collagen types I, III, IV, V and VI at age 4 weeks (left) and 19 weeks (middle) in transgenic pigs, and 19 weeks in wild-type pigs (right). In transgenic pigs, collagen types I, III, IV, V and VI were accumulated in the nodules as early as 4 weeks. Collagen types III, IV and VI were strongly positive. Magnification: 400×.

**Figure 3:**
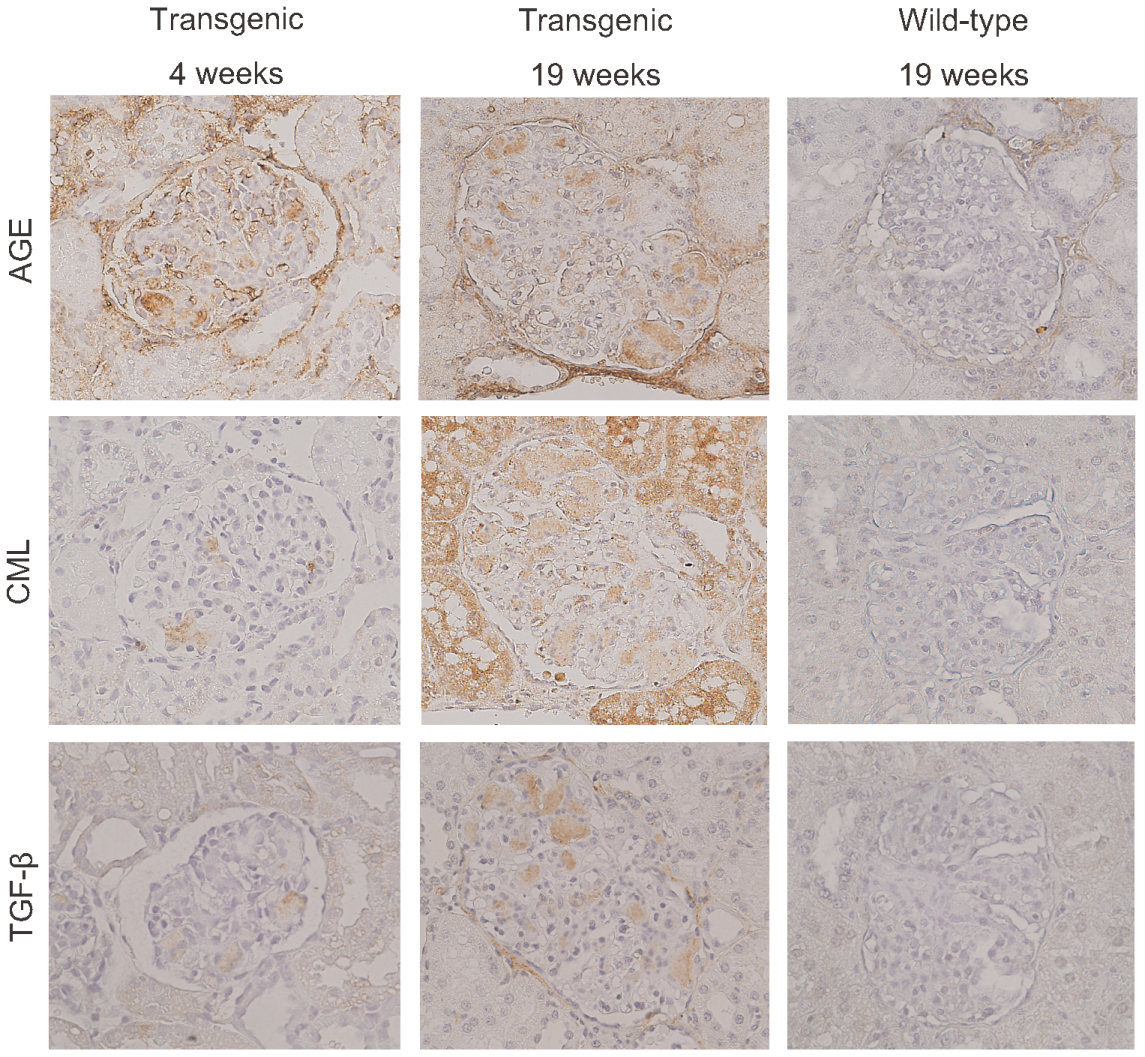
Immunostaining for AGE, CML and TGF-β at age 4 weeks (left) and 19 weeks (middle) in transgenic pigs, and 19 weeks in wild-type pigs (right). In transgenic pigs, AGE, CML and TGF-β accumulated in the glomerular nodules as early as 4 weeks. Magnification: 400×. AGE  =  advanced glycation end product; CML  =  *N^ε^*-carboxymethyl-lysine; TGF-β  =  transforming growth factor-beta.

Immunostaining revealed that the nodules consisted of various types of collagen, including types I, III, IV, V and VI (Figure 2). Collagen types III, IV and VI were present at high concentrations, whereas collagen types I and V were relatively less abundant. AGE, CML and TGF-β1 were also detected in the nodules (Figure 3). AGE tended to be found at the margins of the nodule. CML and TGF-β1 were localized in the nodules in the same patterns as seen in human diabetic nephropathy [20], [21], [22].

To monitor the sequence of nodular formation, monthly kidney biopsies were performed until the age of 10 months. Mesangial expansions were formed as early as 4 weeks of age and contained the abnormal matrices similar to those seen in transgenic pigs at 19 weeks of age (Figure 1A). Thereafter, the matrices expanded gradually with age. Collagen fibers and AGE deposition were exclusively associated from the early evolution to the end of the study period (Figures 2 and 3). Glomerular nodular lesions did not lead to segmental glomerulosclerosis or active adhesion.

Another major histological development was the vacuolization of the cytoplasm of epithelial cells in the proximal tubules, resembling Armanni-Ebstein lesions (Figure S2) [23]. The frequency of mesangiolysis and exudative lesions was low (∼1 per 200 glomeruli). Other diabetic changes normally seen in humans were absent from the pig models, including tubular atrophy, interstitial fibrosis and arteriolar hyalinosis.

### Glomerular nodular lesions consisted of interstitial forms of fibril collagen

**Figure 4:**
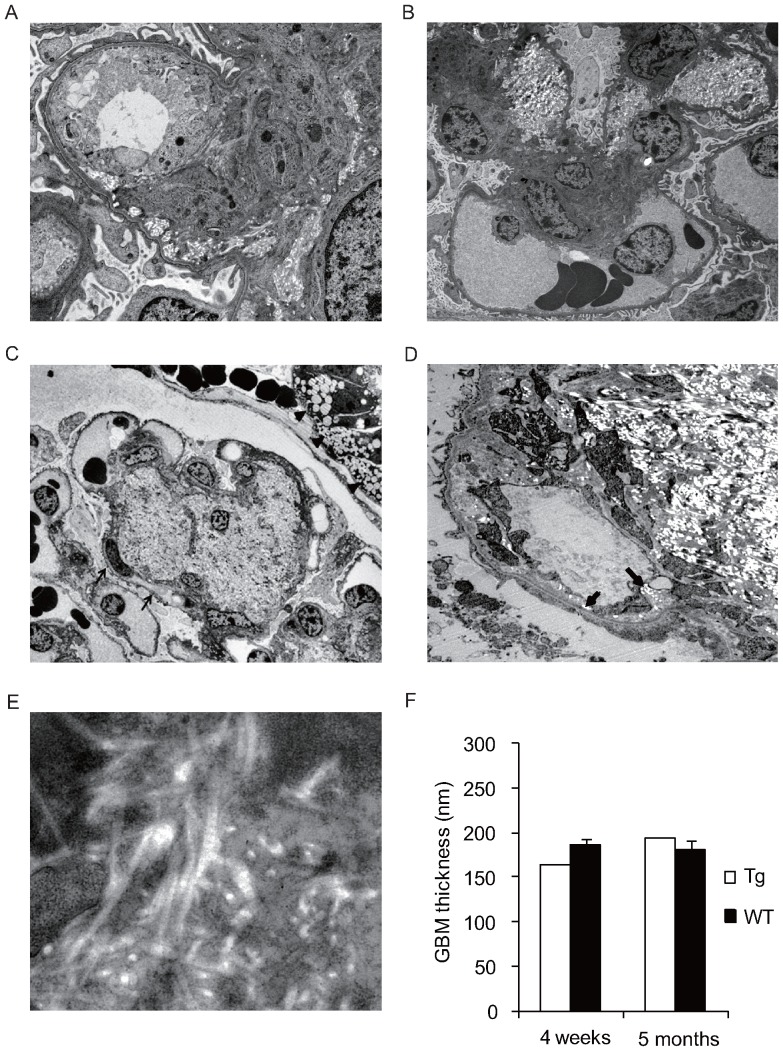
Transmission electron microscopy at age 4 weeks (A,B,E) and 5 months (C,D) in transgenic pigs. **A**) In 4-week-old transgenic pigs, mesangial widening is associated with fiber deposition in the mesangial matrices. Magnification: 2,000×. **B**) Fibers accumulated at mesangial areas, forming early lesion. Magnification: 500×. **C**) At 5 months, established glomerular nodules showed that mesangial areas and capillary lumens are filled with bright fibers (arrows). Vacuolations of proximal tubules were also seen (arrowheads). Magnification: 300×. **D**) Subendothelial widening with loss of endothelial fenestration and mesangial interposition are shown. Note that collagen is also found in the subendothelial spaces (arrows). Magnification: 1,500×. **E**) The nodules consist of fibril collagens with cross striation, indicating interstitial-type forms of collagen fibrils. Magnification: 10,000×. **F**) Thickness of glomerular basement membranes in transgenic pigs was no different from those in wild-type pigs at 4 weeks and 5 months old. Transgenic pigs; n = 1, wild-type pigs; n = 3. Tg  =  transgenic pigs; WT  =  wild-type pigs; GBM  =  glomerular basement membrane.

To determine whether glomerular nodular lesions were associated with the typical diabetic changes found in humans, biopsy specimens from animals at 4 weeks and 5 months of age were visualized by electron microscopy. At 4 weeks, bright fibers began to appear in the mesangial matrices (Figure 4A and B), accompanied by lipid particles and cell debris. At a high magnification, the fibers were seen to closely resemble interstitial types of collagen, being of 46-nm diameter with a 50-nm cross-striation cycle (Figure 4E). These collagens were found predominantly around mesangial cells, suggesting that this was their point of origin (Figure 4A). Within 5 months the fibers had accumulated in the mesangium and had expanded to nodular formations (Figure 4C). This nodule expansion encroached upon capillary lumens and caused them to become occluded. A subendothelial widening, accompanied by a loss of endothelial fenestration and occasional mesangial interposition, was also noted (Figure 4D). The GBM thickness of the transgenic pigs was not different from that of wild-type pigs at both 4 weeks and 5 months of age (4 weeks: 163 nm in transgenic pigs *vs*. 186±10.3 nm in wild-type pigs, 5 months: 194 nm in transgenic pigs *vs*. 181±5.2 nm in wild-type pigs) (Figure 4F).

### Endogenous HNF1α and HNF1β were absent from the glomeruli of wild-type pigs

**Figure 5:**
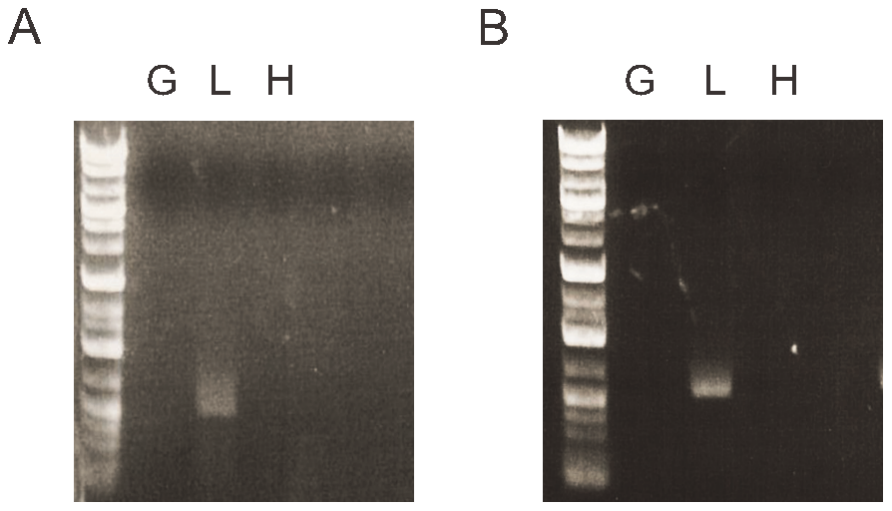
Reverse transcription-PCR for endogenous HNF1α and HNF1β in wild-type pigs at 4 weeks of age. Both HNF1α (**A**) and HNF1β (**B**) were negative in isolated glomeruli. Liver was used as a positive control, and heart as a negative control. G  =  isolated glomeruli; L  =  liver; H  =  heart.

RT-PCR for HNF1α and HNF1β in the glomeruli of wild-type pigs at 4 weeks of age was performed to determine whether insertion of the dominant-negative mutant HNF1αP291fsinsC gene contributed to the development of glomerular nodular lesions by inhibiting endogenous HNF1α or HNF1β function in glomerular cells. Both HNF1α and HNF1β were absent from the isolated glomeruli, but were expressed in the positive control liver tissue (Figure 5A and B). Therefore, the dominant-negative mutant HNF1αP291fsinsC gene insertion did not contribute directly to the glomerular nodular formation in transgenic pigs.

## Discussion and Conclusions

Our pig model carrying a dominant-negative human MODY3 gene is the first to show reproducible diffuse glomerular nodular lesions in a mammalian model of diabetes. The ability to perform repeat kidney biopsies was a great advantage in terms of understanding the *in vivo* morphological events involved in glomerular nodular formation.

Glomerular nodular lesions in our diabetic pigs were characterized by monotonous accumulation of interstitial forms of collagen fibrils in the mesangium. Initially, small nodules were detected as early as 1 month of age and developed diffusely until 10 months of age. Notably, these were basically acellular round nodules without mesangial proliferation, inflammatory infiltrates or mesangiolysis, (cold nodule); this differs from human diabetic nodules. Immunostaining for various collagens revealed predominantly collagen type III, IV, V and VI in our model, similar to in human diabetic nodules [24], [25]. However, our diabetic nodules also exhibited collagen type I deposition, which is unusual in human diabetic nephropathy [24], [25], [26]. Electron microscopy showed a distinct interstitial collagen type, which appeared to be a mixture of types I, III and V collagen, as the main component. This was synthesized in the mesangial cells in the early stage, and tended to expand toward the corresponding capillary lumina, finally resulting in nodular sclerosis.

Although the detailed sequence of events leading to nodular formation, and the structure of the nodules, in this model may not be identical to that in humans with type-2 diabetes, the nodules expressed AGEs from a young age. AGEs are produced by non-enzymatic glycation under hyperglycemia, and glomerular AGE deposition is an important characteristic of nodular morphogenesis in human diabetes [21], [27], [28]. Specifically, CML is the major AGE accumulated in nodular lesions [20], [21]. Glomerular AGEs stimulate extracellular matrix production by mesangial cells through reactive oxygen species (ROS)-promoted TGF-β expression [27], [28], [29], [30]. Glomerular ROS production was caused by AGE-mediated RAGE upregulation or glucose metabolism [27], [28], [30], [31]. In this regard, early onset exclusive AGE deposition and TGF-β1 expression in the nodules of diabetic pigs suggest AGE-mediated collagen synthesis in mesangial cells under a persistent hyperglycemic condition. The differences in nodular morphogenesis and its collagen composition between our model and human diabetic nephropathy suggest that the mesangial cellular response in these species is different under diabetic conditions. Several reports of nodular sclerosis in the diabetic rodent model support this explanation.

In addition to the mesangial changes under hyperglycemia, our model suggests the involvement of unique hemodynamic factors in nodular morphogenesis. Glomerular hyperfiltration or hypertrophy promotes diabetic nephropathy; however, whether glomerular hemodynamic effects accelerate the formation of diabetic nodules in humans remains controversial. Accordingly, the present study showed that glomerular nodular lesions in diabetic pigs were localized predominantly in the deep cortex. Notably, glomeruli were significantly larger in the deep cortex of diabetic pigs compared to in that of wild-type pigs, but were unchanged in the superficial cortex of both groups. These observations suggest that glomerular hemodynamic effects also promote formation of glomerular nodular lesions in diabetic pigs. Similarly, diabetic nephropathy was accelerated in eNOS-knockout mice, attenuated by improvement of eNOS activity in *db/db* mice [32], [33], and antihypertensive therapy alone significantly suppressed the development of nodular lesions and mesangiolysis in diabetic eNOS-knockout mice [34]. Based on these reports and our current findings, our results suggest the involvement of glomerular hypertension in nodular lesion formation in diabetes. Therefore, prominent glomerular hyperfiltration and hypertrophy may be the basis of glomerular nodule development in our diabetic pig model.

The inserted dominant-negative human MODY gene might have stimulated mesangial matrix synthesis by inhibiting endogenous HNF1α or HNF1β function, regardless of the diabetic milieu. Typically, HNF1α functions as a homodimer or a heterodimer with the structurally related protein HNF1β [14], [15], [35]. Thus, a dominant-negative mutant HNF1αP291fsinsC should inhibit HNF1α or HNF1β by forming an inactive heterodimer at the site of endogenous HNF1α or HNF1β expression. The RT-PCR study confirmed that mutant HNF1αP291fsinsC could not interact with endogenous HNF1α and HNF1β in the glomeruli of transgenic pigs. This supports the notion that the diabetic milieu, but not the genetic alteration, promotes glomerular nodular formation in the pig model.

Our diabetic pig model lacks several diabetic renal features characteristic of human diabetic nephropathy; e.g., proteinuria, GBM thickening, exudative lesions, tubular atrophy, interstitial fibrosis and arteriolar hyalinosis. Furthermore, the glomeruli did not undergo glomerulosclerosis. These results suggest that our model does not accurately reproduce human diabetic nephropathy, even in pigs carrying the human MODY3 gene. In addition to the species difference in the cellular response to hyperglycemia, a possible explanation for this discrepancy is that we were unable to monitor the histology for a sufficiently long period because due to the relatively short lifespan of the pigs. In addition, the mechanism of nodular formation is considered to be different from that of other diabetic kidney lesions. Nevertheless, this pig model indicated that glomerular nodules could form independently of diabetic complications.

In conclusion, this was the first report of distinct and reproducible glomerular nodular lesions in transgenic pigs carrying a dominant-negative HNF1α mutation of the human MODY3 gene. Although there were several differences compared to the pathology of human glomerular nodular lesions, the somewhat acute and constitutive formation of nodules in the mammalian models might provide information that will facilitate identification of the principal mechanism underlying glomerular nodular formation.

## Supporting Information

Figure S1 **Body weight and diabetic parameter changes over time.** **A**) Body weight was lower in transgenic pigs than in wild-type pigs. **B**) Plasma glucose was at a high level in transgenic pigs. **C**) 1,5-Anhydroglucitol was at a low level in transgenic pigs. Tg  =  transgenic pigs (n = 1); WT  =  wild-type pigs (up to 6 months of age, n = 3; 6–10 months of age, n = 1).
(TIF)

Figure S2 **Armanni-Ebstein lesions in diabetic pigs at 19 weeks of age.** Transgenic pigs revealed vacuolation of proximal tubules known as Armanni-Ebstein lesions. Note that distal tubules and the collecting duct are intact. **A**) Magnification: 100×. **B**) Magnification: 400×.
(TIF)
