# Supplementary figures and images for: Diffuse Glomerular Nodular Lesions in Diabetic Pigs Carrying a Dominant-Negative Mutant Hepatocyte Nuclear Factor 1-Alpha, an Inheritant Diabetic Gene in Humans
Source: PLoS One. 2014 Mar 19;9(3):e92219. doi: 10.1371/journal.pone.0092219 (PMC3960229; doi:10.1371/journal.pone.0092219)

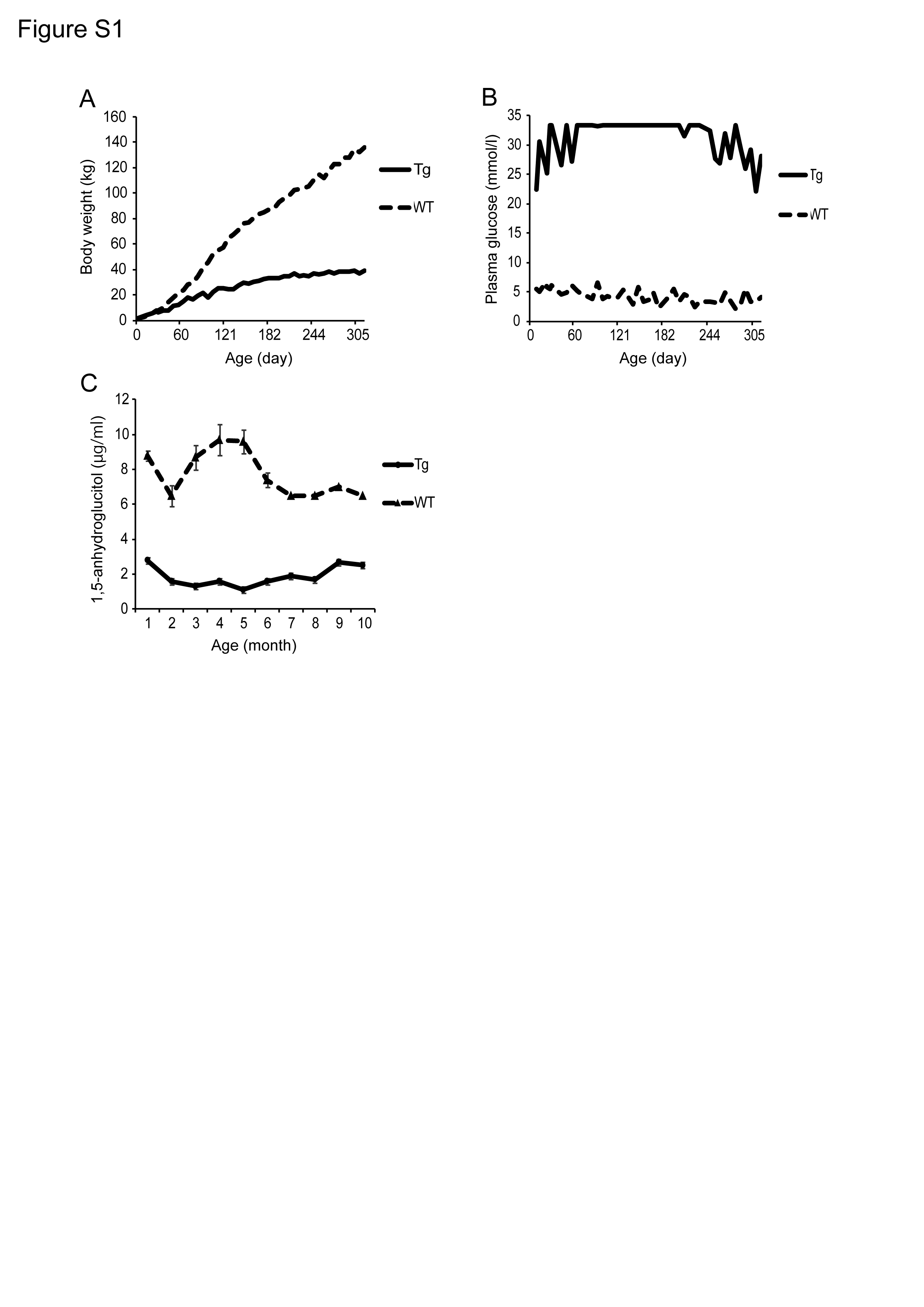

Supplement: Figure S1 — Body weight and diabetic parameter changes over time. A) Body weight was lower in transgenic pigs than in wild-type pigs. B) Plasma glucose was at a high level in transgenic pigs. C) 1,5-Anhydroglucitol was at a low level in transgenic pigs. Tg = transgenic pigs (n = 1); WT = wild-type pigs (up to 6 months of age, n = 3; 6–10 months of age, n = 1). (TIF) [file pone.0092219.s001.tif]

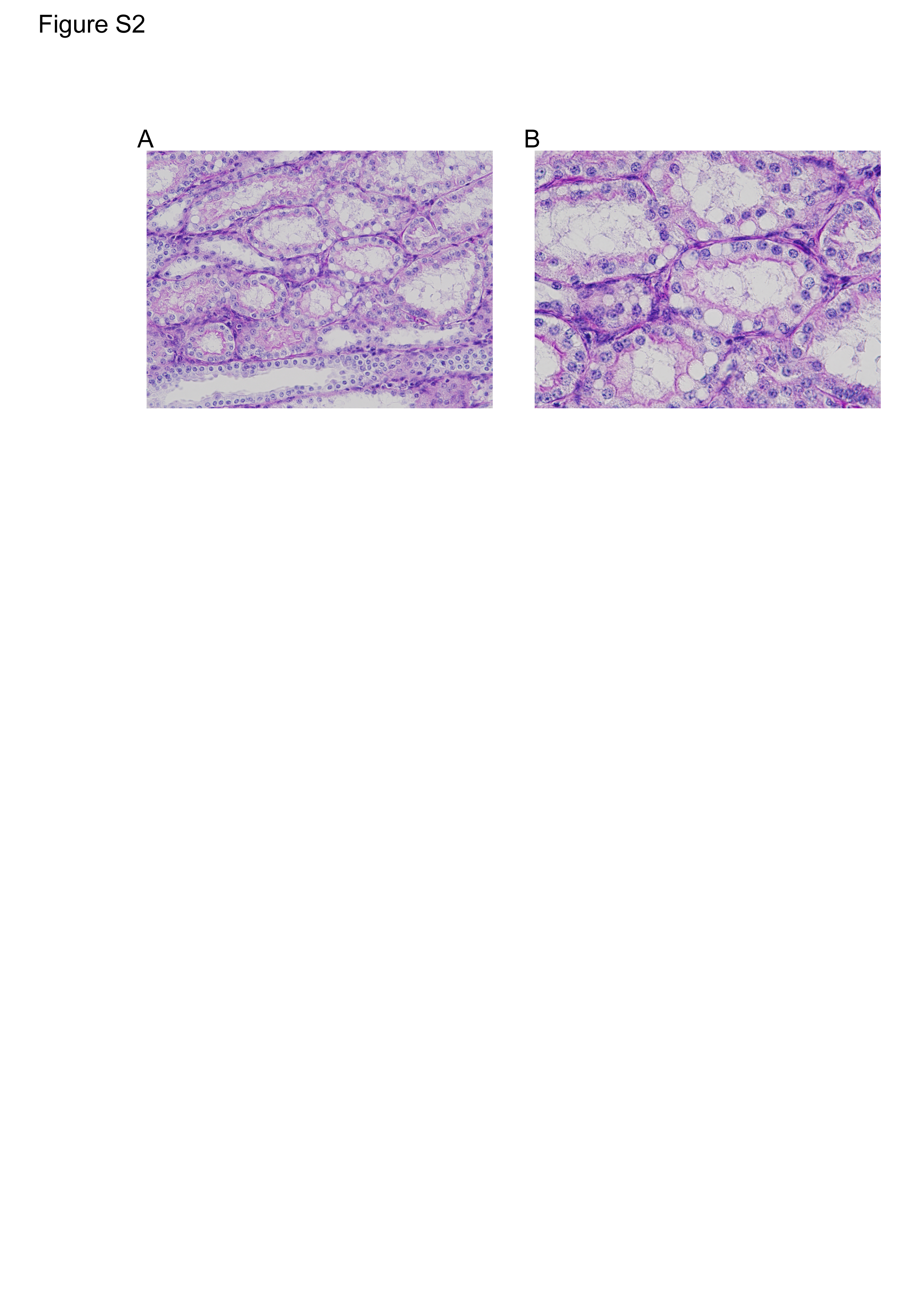

Supplement: Figure S2 — Armanni-Ebstein lesions in diabetic pigs at 19 weeks of age. Transgenic pigs revealed vacuolation of proximal tubules known as Armanni-Ebstein lesions. Note that distal tubules and the collecting duct are intact. A) Magnification: 100×. B) Magnification: 400×. (TIF) [file pone.0092219.s002.tif]
